# Identification of shared lactylation-related gene signatures between osteoporosis and chronic kidney disease

**DOI:** 10.3389/fcell.2025.1719273

**Published:** 2025-12-11

**Authors:** Dong Liu, Ying Xia, Hui Wang, Kaiqiang Sun, Shunmin Wang, Yaping Yu, Xiaojie Dai

**Affiliations:** 1 Department of Urology, Shanghai Changzheng Hospital, Shanghai, China; 2 Department of Orthopedic Surgery, Changzheng Hospital, Navy Medical University, Shanghai, China

**Keywords:** osteoporosis, chronic kidney disease, lactylation, nomogram, machine learning

## Abstract

**Objectives:**

Chronic kidney disease (CKD) and osteoporosis (OP) frequently coexist, yet their shared molecular pathogenesis remains incompletely characterized. We sought to identify diagnostic gene signatures for CKD and OP through integrated bioinformatics analysis, developing machine learning-based predictive models and clinical nomograms.

**Methods:**

Transcriptomic datasets (GSE104948, GSE104954, GSE56814, GSE56815) were normalized and batch-corrected. Differential expression analysis identified cross-disease signatures, followed by gradient boosting machine (GBM) and random forest modeling. Nomograms were constructed and validated via ROC curves and calibration plots. Findings were corroborated through immune correlation analyses and an ovariectomy (OVX) mouse model with/without CKD.

**Results:**

Shared differentially expressed genes (DEGs) revealed six hub genes (MSN, PCBP2, CHERP, EMG1, RALYL, ALDH1A1) with significant expression differences. The GBM model achieved robust predictive performance. The CKD nomogram demonstrated excellent discrimination (AUC discovery = 0.8915; validation = 0.9837), while the OP nomogram showed moderate discriminatory capacity (AUC discovery = 0.8085; validation = 0.65). Murine model studies confirmed CKD synergistically exacerbates OP progression.

**Conclusion:**

We establish a high-accuracy CKD diagnostic nomogram and identify critical gene signatures common to CKD and OP pathogenesis. While the OP model requires refinement, these findings provide clinically actionable tools for precision diagnosis and illuminate molecular mechanisms linking renal and skeletal pathology.

## Introduction

Chronic kidney disease (CKD) is a progressive and irreversible condition characterized by multisystem involvement, including disruptions in mineral and bone metabolism (Mace et al., 2021). Increasing evidence points to a close association between CKD and osteoporosis (OP), a skeletal disorder marked by reduced bone mass and increased fracture risk (Bover et al., 2021). Epidemiological studies have revealed that the prevalence of OP is significantly elevated in CKD patients, especially in those at more advanced stages of renal dysfunction (Titan et al., 2020). For example, approximately 15.4% of individuals with stage 3 CKD have been diagnosed with OP, a proportion substantially higher than that observed in the general population (Razia et al., 2022; Chiang et al., 2022). In addition, the progression of CKD is frequently accompanied by mineral and bone disorders (CKD-MBD), secondary hyperparathyroidism, and abnormalities in calcium-phosphate homeostasis, all of which contribute to impaired bone quality and fragility fractures (Pawlak et al., 2021). Besides, the presence of OP in CKD patients is associated with increased fracture incidence and adverse clinical outcomes, including loss of mobility and diminished quality of life (Jiang et al., 2020).

Meanwhile, the Yellow Emperor’s Inner Canon records: Prolonged exertion with heat exposure induces excessive thirst. This triggers internal conflict of yang qi, leading to heat lodging in the kidneys. As the water-organ, the kidneys fail to restrain fire (pathogenic heat), resulting in desiccated bones and depleted marrow. Consequently, the legs cannot support the body, manifesting as bone flaccidity (Liu et al., 2020; Tian et al., 2024) This illustrates that yin-yang imbalance in the kidneys underlies osteoporosis pathogenesis. In this context, osteoblasts (OBs), responsible for bone formation—are categorized as yin-type cells, while osteoclasts (OCs), mediating bone resorption, are yang-type cells (Zhu et al., 2023). Physiologically, the dynamic equilibrium between OBs and OCs maintains skeletal homeostasis. However, osteoporosis arises from disruption of OB-OC homeostasis (Zhang et al., 2025).

These findings demonstrate a bidirectional, multifactorial relationship between CKD and OP. Nevertheless, the pathophysiological basis linking renal dysfunction to bone loss remains incompletely characterized; further investigation is required to define the molecular and systemic interactions underlying this comorbidity. Elucidating these mechanisms is essential for developing targeted interventions to improve prevention and clinical management in affected populations.

Lactylation, a recently identified post-translational modification derived from lactate metabolism, has emerged as a crucial regulatory mechanism linking cellular metabolic states to gene expression and protein function (Xin et al., 2022). Lactylation occurs on lysine residues of histones and non-histone proteins, thereby influencing chromatin accessibility and cellular responses to metabolic cues (Drapela et al., 2022). In the context of CKD, lactylation has been implicated in the regulation of inflammation, fibrosis, and metabolic reprogramming (Wang et al., 2024a). Studies have shown that the accumulation of lactate under hypoxic or inflammatory conditions enhances histone lactylation, promoting the transcription of profibrotic and inflammatory genes (Wang et al., 2024a; Zhu et al., 2024). Furthermore, lactylation modulates macrophage polarization, which is pivotal in CKD progression (Xiang et al., 2025). Similarly, in OP, lactylation is gaining attention as a potential modulator of bone homeostasis. Recent evidence suggests that lactylation affects osteoblast differentiation and osteoclast activity by altering key signaling pathways (Wu et al., 2023; Li et al., 2025). Moreover, lactylation may coordinate the cellular response to oxidative stress and energy metabolism, processes intricately linked to bone remodeling (Chen et al., 2025; Li et al., 2022). We therefore hypothesize that lactylation may act as a key metabolic bridge linking the dysregulated cellular environments of CKD and OP. In CKD, hypoxia and inflammation drive lactate accumulation, while in OP, altered cellular metabolism in the bone microenvironment may similarly elevate lactate levels. Thus, we propose that this shared metabolic perturbation, elevated lactate, converges to drive aberrant protein lactylation, which in turn dysregulates critical genes and pathways common to both disease states.

Therefore, this study aims to test this hypothesis by defining the shared lactylation-related gene signatures between CKD and OP through integrative bioinformatics analysis of public gene expression databases. By defining shared gene signatures and molecular pathways, we elucidate potential common mechanisms underlying CKD-OP comorbidity. These insights inform novel therapeutic strategies to mitigate disease progression in comorbid patients, ultimately improving clinical outcomes and health-related quality of life.

## Methods and materials

### Data Collection and preprocessing

Gene expression datasets (GSE104948, GSE104954, GSE56814, GSE56815) were accessed from the Gene Expression Omnibus (GEO). GSE104948 (n = 196) and GSE104954 (n = 195) were generated from glomerular transcriptome from European Renal cDNA Bank subjects and living donors, which was also included under previous GEO submissions-GSE47183 (chronic kidney disease samples), and GSE32591 (IgA nephropathy samples) (Grayson et al., 2018). GSE37171 was used to study gene expression changes in uremia using whole genome microarray analysis of peripheral blood from subjects with end-stage renal failure (n = 63) and healthy controls (n = 20) (Scherer et al., 2013). GSE56814 (n = 73) and GSE56815 (n = 80) included the microarray analyses of circulating monocytes from pre- and postmenopausal females with low or high bone mineral density (BMD) (Zhou et al., 2018a; Zhou et al., 2018b). GSE7429 included the gene expression patterns of circulating B cells in blood from 20 postmenopausal females with low or high bone mineral density (BMD) (Xiao et al., 2008).

Selection criteria included relevance to study objectives and experimental design consistency. Raw data were processed in R (v4.3.0) using the sva package’s ‘ComBat’ function for batch effect correction to mitigate cross-dataset technical artifacts. Subsequent normalization employed the Robust Multiarray Average (RMA) method to stabilize variance. Quality control included principal component analysis (PCA) and boxplot visualization to evaluate batch correction efficacy and normalization performance.

### Differential expression analysis

Differential expression analysis identified genes with statistically significant changes between experimental groups using the limma package in R. This approach employs empirical Bayes moderation to enhance variance estimation in limited-sample studies. Result interpretation was facilitated through: (1) Volcano plots visualizing significance versus fold-change distributions, and (2) Hierarchically clustered heatmaps displaying expression profiles of top-ranked differentially expressed genes (DEGs) across samples.

### Machine learning algorithms for predictive modeling

We implemented multiple machine learning algorithms in R to construct predictive models and identify key gene signatures. Gradient Boosting Machine (GBM) - an ensemble method that iteratively builds decision trees to minimize prediction error - was employed for enhanced classification performance. A hybrid approach combining stepwise logistic regression (feature selection via Akaike Information Criterion) with Random Forest (RF) refined feature selection and improved model stability. Model performance was evaluated using Receiver Operating Characteristic (ROC) curves, with discriminatory capacity quantified by the Area Under the Curve (AUC).

### Biomarker evaluation

To evaluate the diagnostic potential of the identified candidate genes, their expression levels were compared between disease and control groups using normalized transcriptomic data. Statistical analysis was conducted to assess the significance of differential expression, and genes demonstrating consistent and marked expression differences were prioritized. ROC curve analysis was performed for each top-ranking gene to determine its individual diagnostic performance. The AUC was calculated to quantify sensitivity and specificity, with higher AUC values indicating greater discriminatory power.

### Nomogram construction and validation

A nomogram model was constructed based on the most predictive gene signatures identified through prior machine learning analyses. The nomogram was developed using a multivariate logistic regression model. The regression coefficients (β) from this model were used to assign points to each gene signature. Specifically, the point assignment for each level of a gene’s expression was proportional to its β coefficient, with a higher weight (more points) indicating a stronger contribution to the final risk score for disease prediction. The model integrated these gene markers to estimate disease risk. To evaluate the predictive performance of the nomogram, ROC curve analysis was conducted in both the discovery and independent validation cohorts, with the AUC used to quantify diagnostic accuracy. Calibration curve analysis was employed to assess the agreement between predicted probabilities and observed outcomes, thereby evaluating the model’s reliability. Additionally, Decision Curve Analysis (DCA) was performed to estimate the clinical net benefit across a range of threshold probabilities, providing insight into the model’s utility in clinical decision-making. Clinical impact curves were further generated to visualize the potential population-level benefit of the nomogram in stratifying individuals based on risk.

### Immune cell correlation analysis

To investigate the immunological relevance of the identified candidate genes, immune cell correlation analysis was conducted to estimate the relative proportions of immune cell types within the gene expression profiles. Multi-parametric correlation analyses were performed to evaluate the associations between candidate gene expression levels and various immune cell subsets.

### Mouse model establishment

To investigate the combined effects of CKD and estrogen deficiency on bone metabolism, a CKD and ovariectomy (CKD + OVX) mouse model was established. Female mice were randomly assigned to three groups: Sham-operated controls (Sham), ovariectomized (OVX), and CKD combined with OVX (CKD + OVX). CKD was induced through 5/6 nephrectomy in two stages under aseptic conditions, as previously described (Nakano et al., 2019), to mimic progressive renal dysfunction. Two weeks following CKD induction, bilateral ovariectomy was performed to simulate estrogen deficiency. Sham mice underwent identical surgical procedures without removal of renal tissue or ovaries. All animals were housed under standard laboratory conditions with free access to food and water. Animal protocols were approved by our institution ethic committee, and all procedures were conducted in accordance with ethical guidelines for animal experimentation.

### Micro-computed tomography analysis

Micro-computed tomography (micro-CT) was employed to evaluate trabecular bone microarchitecture in the lumbar vertebrae and distal femurs of experimental mice. Specimens were scanned using a high-resolution micro-CT system (Bruker, Skyscan1276) for quantitative disc height evaluation.

### Histological analysis and tartrate-resistant acid phosphatase staining

Histological analysis was conducted to assess trabecular bone integrity and osteoclast activity in lumbar vertebrae and femoral bones. Bone specimens were fixed in 4% paraformaldehyde, decalcified in 10% EDTA, embedded in paraffin, and sectioned at 5 μm thickness. Hematoxylin and eosin (H&E) staining was performed to evaluate general bone morphology and trabecular structure. For osteoclast detection, tartrate-resistant acid phosphatase (TRAP) staining was carried out using a commercially available TRAP staining kit according to the manufacturer’s instructions. TRAP^+^ multinucleated cells were identified as osteoclasts.

### Statistical analysis

Sample sizes for animal experiments were determined based on established experimental protocols and effect sizes consistently observed in prior studies from our laboratory and relevant literature utilizing similar disease models and outcome measures (Sun et al., 2025; Qin et al., 2024; Mao et al., 2023). This approach ensured that the sample size was adequate to detect statistically significant and biologically relevant effects. All experimental assessments and data analyses were conducted in a blinded manner with respect to group allocation. Data are presented as mean ± standard deviation (SD). All statistical analyses were performed using GraphPad Prism 9 (GraphPad Software Inc., United States). To assess homogeneity of variance among groups, an F-test was performed. Normality of data distribution was evaluated using the Shapiro-Wilk or D’Agostino-Pearson test. When comparing two groups, the Student’s unpaired t-test was applied. Comparisons among more than two groups were conducted using one-way ANOVA followed by Tukey’s post hoc test. For analyses involving two independent variables (e.g., OVX and CKD), two-way ANOVA with Tukey’s post hoc test was used. The number of animals (n) used in each group is indicated in the figure legends and represents independent biological replicates. A p-value <0.05 was considered statistically significant.

## Results

### Data processing and integration and differential gene expression profiling

The analytical workflow for CKD and OP followed a systematic approach. Raw transcriptomic datasets (GSE104948, GSE104954, GSE56814, and GSE568145) were retrieved from the GEO database. Following preprocessing, batch effects were systematically removed to mitigate technical variability. Subsequent normalization yielded integrated datasets for CKD and OP, enabling cross-dataset comparisons. PCA was conducted independently for CKD (Supplementary Figure 1A) and OP (Supplementary Figure 1B) to evaluate inter-dataset heterogeneity post-batch correction. For CKD, the first two principal components (Dim1: 72.2%, Dim2: 4.4%) explained the majority of variance. Similarly, OP demonstrated Dim1 (61.5%) and Dim2 (11.7%) capturing the largest proportion of variability.

**FIGURE 1:**
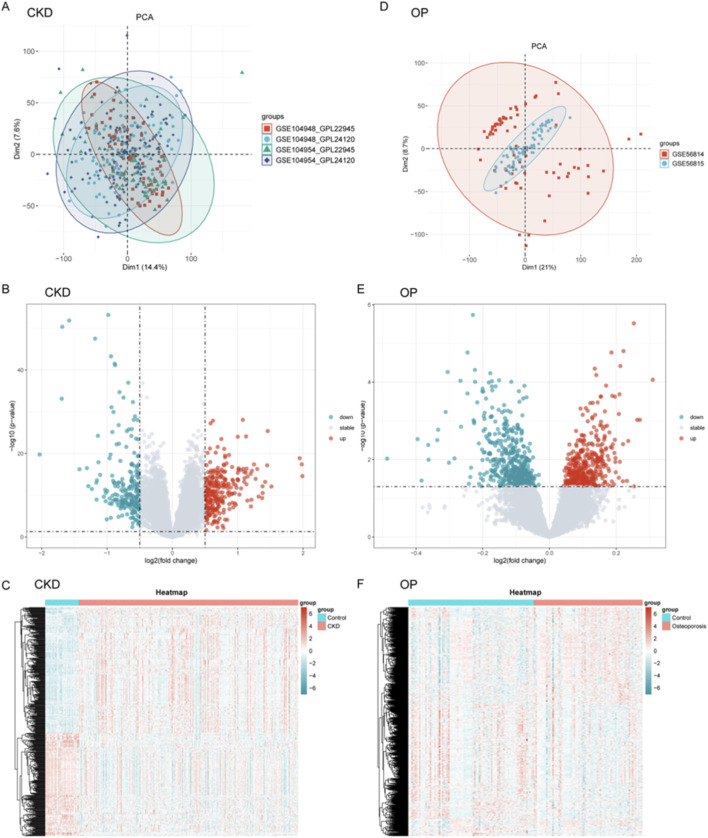
Data processing, integration, and differential gene expression profiling for CKD and OP. **(A–D)** Principal component analysis (PCA) was performed for CKD **(A)** and OP **(D)** datasets to assess inter-dataset heterogeneity post-batch correction. **(B–E)** Volcano plots were generated to visualize differential gene expression between CKD and OP. **(C–F)** Heatmaps further illustrate disease-specific expression signatures, showing the clustering of genes in CKD and OP cohorts.

Integrated transcriptomic datasets from CKD (GSE104948, GSE104954) and OP (GSE56814, GSE56815) cohorts underwent batch effect correction and normalization. PCA revealed distinct clustering patterns between various datasets (Figures 1A,D). Differential expression analysis with volcano plots highlighted CKD-associated gene expression upregulation and OP-linked suppression (Figures 1B,E). Heatmaps further visualized disease-specific expression signatures (Figures 1C,F).

### Predictive model construction and key gene identification

Venn diagrams delineated molecular intersections between CKD and OP pathogenesis (Supplementary Figure 2A). A total of 562 differentially expressed genes (DEGs) were shared between CKD and OP, while 327 and 7,043 genes were unique to OP and CKD, respectively (Supplementary Figure 2A). Integration of lactylation-associated genes further revealed a core set of 15 genes common to all three categories (CKD_DEGs, OP_DEGs, and lactylation genes), suggesting shared epigenetic regulation (Supplementary Figure 2B; Supplementary Table S1).

**FIGURE 2:**
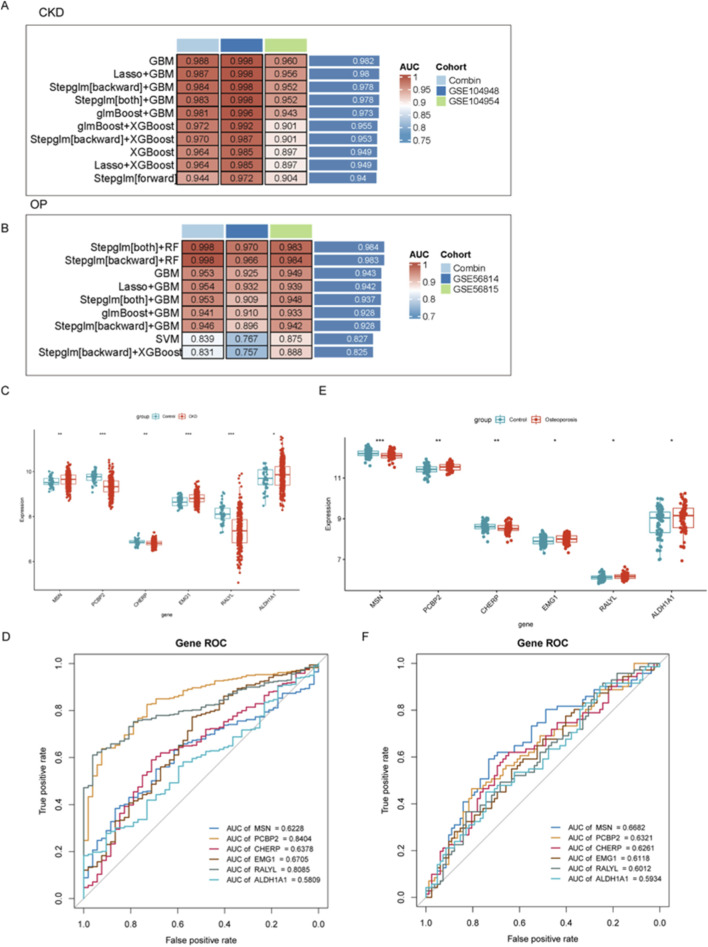
Evaluation of diagnostic gene expression profiles and machine learning models for CKD and OP. **(A,B)** Performance of machine learning models in predicting CKD and OP outcomes. **(C–E)** Gene expression differences between disease and control groups for the top six key genes (MSN, PCP2P, CHERP, EMG1, RALYL, and ALDH1A1). **(D–F)** ROC analyses assessing the diagnostic potential of key biomarkers in CKD and OP.

To evaluate the diagnostic potential of gene expression profiles in CKD and OP, multiple machine learning algorithms were employed to construct predictive models across combined and independent cohorts. As shown in Figures 2A,B, GBM-based models demonstrated consistently superior performance in both disease contexts. In CKD, the GBM approach achieved the highest AUC value in the Combin cohort (AUC = 0.988), as well as in independent validation cohorts GSE104948 and GSE104954. Similarly, in OP, integrated models such as stepwise regression with random forest (Steplm [both]+RF and Steplm [backward]+RF) achieved robust discrimination performance (AUC = 0.998 in Combin; AUC = 0.983–0.984 in GSE56814 and GSE56815 cohorts), suggesting the reliability and generalizability of these models across datasets. In conclusion, we primarily highlighted the GBM for CKD due to its consistently superior AUC across both discovery and independent validation cohorts, indicating exceptional predictive robustness for this condition (Figure 2A). For OP, the hybrid stepwise-RF model was emphasized not only for its high performance but also for its enhanced feature selection process (Figure 2B). The stepwise regression prior to RF modeling provided a more stringent and interpretable subset of genes, which was deemed advantageous for elucidating the potentially more complex and multifactorial pathogenesis of OP.

To further identify and validate diagnostic gene signatures, the expression levels of the top six key genes (MSN, PCP2P, CHERP, EMG1, RALYL, and ALDH1A1) were compared between disease and control groups. In both CKD and OP, the expression of these genes showed significant differences between groups, indicating potential roles in disease pathogenesis. Notably, CHERP was significantly downregulated in both CKD and OP and EMGA and ALDH1A1 were upregulated in both CKD and OP (Figures 2C,E). Although the expression levels of other genes were also remarkably changed, their expression models were not consistent in CKD and OP, revealing distinct gene expression patterns associated with disease state (Figures 2C,E). To assess the discriminative capacity of these biomarkers, receiver operating characteristic (ROC) analyses were conducted. As illustrated in Figure 2D, PCBP2 exhibited the highest diagnostic accuracy for CKD with an AUC of 0.8404, followed by RALYL (AUC = 0.8085). ROC analysis was also carried out in the OP cohort (Figure 2F), where MSN showed the best performance (AUC = 0.6682).

### Construction and validation of a diagnostic nomogram model for CKD based on gene signatures

**FIGURE 3:**
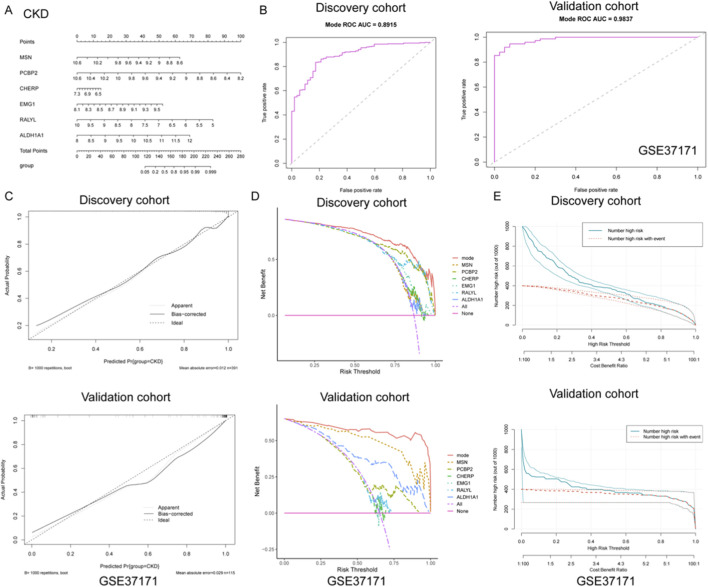
Construction and validation of a diagnostic nomogram model for CKD based on gene signatures. **(A)** A nomogram was developed using six key gene signatures (MSN, PCBP2, CHERP, EMG1, RALYL, and ALDH1A1) to predict CKD risk. The model provides a visual scoring system that translates gene expression levels into a total point score, facilitating risk assessment for CKD. **(B)** ROC curve analysis of the nomogram model in both discovery and validation cohorts. **(C)** Calibration curves for the discovery and validation cohorts, showing the close alignment between predicted and actual CKD outcomes. **(D)** DCA illustrating the net benefits of the full nomogram model versus individual gene predictors or no intervention across a range of risk thresholds. The nomogram model consistently yielded higher net benefits, highlighting its clinical utility. **(E)** Clinical impact curves for both the discovery and validation cohorts, displaying the relationship between predicted high-risk individuals and actual CKD events across varying thresholds. The strong alignment between predicted and observed outcomes underscores the nomogram’s ability to effectively stratify risk in clinical practice.

To develop a robust diagnostic tool for CKD, a nomogram model was constructed based on six gene signatures (MSN, PCBP2, CHERP, EMG1, RALYL, and ALDH1A1) derived from integrated machine learning approaches. As shown in Figure 3A, the nomogram provides a visualized scoring system that translates gene expression levels into total points for predicting CKD risk. To evaluate the model’s discrimination ability, ROC curves were plotted for both discovery and validation cohorts. The model achieved excellent diagnostic performance, with an AUC of 0.891 (sensitivity = 0.827, specificity = 0.835) in the discovery set and an AUC of 0.984 (sensitivity = 0.925, specificity = 0.947) in the independent GSE37171 validation cohort (Figure 3B), indicating high predictive accuracy and generalizability.

Further calibration analyses were performed to assess the agreement between predicted and actual outcomes. As shown in Figure 3C, the calibration curves of both cohorts closely approximated the ideal diagonal, with low mean absolute errors (0.012 in discovery; 0.029 in validation), demonstrating that the nomogram provides unbiased and reliable probability estimates for CKD occurrence. In addition, decision curve analysis (DCA) was utilized to determine the clinical utility of the nomogram and individual gene predictors. As illustrated in Figure 3D, the full model (mode) consistently yielded higher net benefits across a wide range of risk thresholds compared to any single gene or no intervention, underscoring its practical value in guiding clinical decision-making. Moreover, clinical impact curves were generated to visualize the model’s population-level implications. As presented in Figure 3E, both the number of individuals classified as high-risk and those truly affected by CKD were plotted across varying cost-benefit thresholds. The strong alignment between predicted and actual events further validated the model’s capacity for effective risk stratification in clinical settings. Collectively, these findings support the potential application of this nomogram as a reliable diagnostic tool for CKD, offering both precision and clinical utility.

### Development and validation of a gene-based nomogram model for osteoporosis diagnosis

**FIGURE 4:**
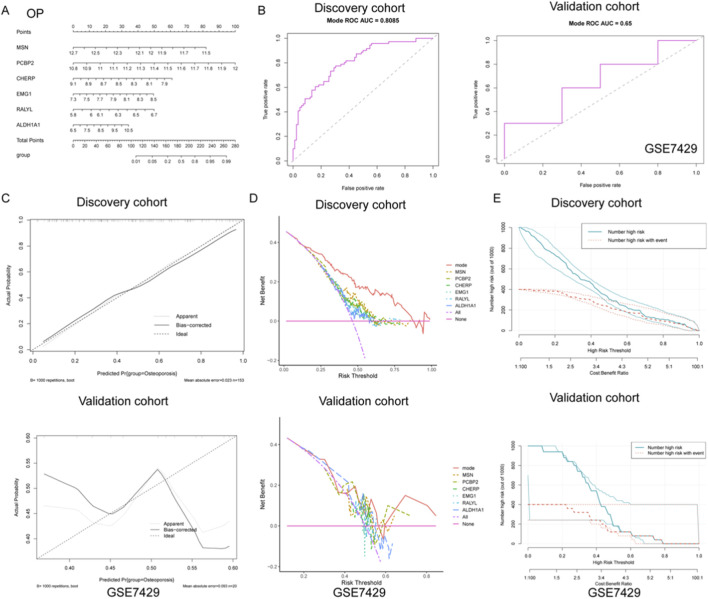
Development and validation of a diagnostic nomogram model for OP based on gene expression signatures. **(A)** A nomogram was constructed using six key genes (MSN, PCBP2, CHERP, EMG1, RALYL, and ALDH1A1) to integrate individual gene expression levels into a total risk score for predicting OP occurrence. **(B)** ROC curve analysis demonstrated strong diagnostic performance of the nomogram in the discovery cohort, while only moderate discriminative ability was observed in the external validation cohort GSE7429. **(C)** Calibration curves in the discovery cohort revealed high agreement between predicted and observed probabilities, suggesting good model calibration. In contrast, the validation cohort showed deviation from the ideal diagonal, indicating reduced calibration accuracy. **(D)** DCA showed that the nomogram model provided the greatest net clinical benefit over a wide range of threshold probabilities in the discovery cohort. **(E)** Clinical impact curves revealed that in the discovery cohort, the predicted number of high-risk individuals closely matched the actual number of OP cases across risk thresholds, supporting the model’s clinical relevance.

To further explore the diagnostic value of the identified gene signatures in OP, a predictive nomogram model was constructed based on the six hub genes (MSN, PCBP2, CHERP, EMG1, RALYL, and ALDH1A1), integrating individual gene scores to estimate the probability of OP occurrence (Figure 4A). The diagnostic efficiency of the nomogram was evaluated using ROC curve analysis. In the discovery cohort, the model demonstrated robust discriminatory power with an AUC of 0.808 (sensitivity = 0.695, specificity = 0.775), indicating strong classification ability (Figure 4B). However, the model exhibited only moderate predictive capacity in the external validation cohort (GSE7429), with an AUC of 0.650 (sensitivity = 0.500, specificity = 0.800) (Figure 4B).

To further assess the reliability of the nomogram predictions, calibration curves were plotted to compare the predicted versus actual probabilities of osteoporosis. The calibration plot in the discovery cohort revealed a strong concordance between predicted and observed values, with a low mean absolute error (0.023), suggesting good calibration (Figure 4C). In contrast, the validation cohort exhibited noticeable deviation from the ideal line, indicating reduced calibration accuracy in the external dataset (Figure 4C). In addition, DCA was applied to determine the clinical net benefit of the nomogram relative to each single gene and the absence of intervention. As shown in Figure 4D, the nomogram (mode) yielded the highest net benefit across a broad range of risk thresholds in the discovery cohort, while its performance was less consistent in the validation cohort, although it still outperformed individual genes such as RALYL and ALDH1A1. Moreover, clinical impact curves were plotted to visualize the potential clinical utility of the model across varying high-risk thresholds. In the discovery cohort, the number of individuals identified as high-risk closely mirrored the actual number of positive cases, underscoring the model’s population-level applicability (Figure 4E). Conversely, in the validation cohort, the disparity between predicted and actual high-risk cases was more evident, again reflecting moderate generalizability (Figure 4E). Collectively, these findings suggest that while the nomogram demonstrates strong diagnostic capacity in the training set, further optimization is needed to enhance its robustness and external applicability for osteoporosis prediction.

### Correlation profiling of immune cell types and candidate genes in CKD

**FIGURE 5:**
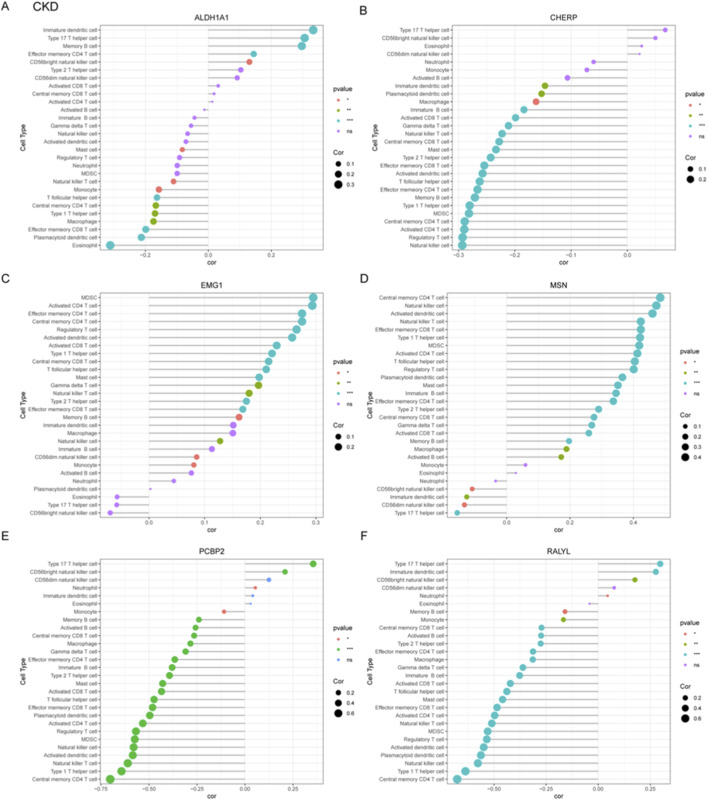
Correlation profiling between diagnostic gene signatures and immune cell infiltration patterns in CKD. **(A–F)** Correlation analyses were performed to assess the associations between six candidate genes and immune cell subtypes, which include ALDH1A1 **(A)**, CHERP **(B)**, EMG1 **(C)**, MSN **(D)**, PCBP2 **(E)**, and RALYL **(F)**.

To systematically investigate immune-pathogenic mechanisms in CKD progression, we conducted comprehensive correlation analyses between six functionally significant genes (ALDH1A1, CHERP, EMG1, MSN, PCBP2, RALYL) and various immune cell subtypes through multi-parametric visualization. ALDH1A1 showed significant negative correlations with immature dendritic cell and type 17 T helper cell, but positive correlation with effector memory CD8^+^ T cell (Figure 5A). Conversely, PCBP2 exhibited strong positive correlations with central memory CD4^+^ T cell (Figure 5E). The MSN gene demonstrated strong positive correlation with type 17 T helper cell but negative correlation with central memory CD4^+^ T cell (Figure 5D). CHERP positively correlated with natural killer cell, RALYL with central memory CD4^+^ T cell, and EMG1 negatively correlated with MDSCs (myeloid-derived suppressor cells) (Figure 5B,C,D).

### Immune-gene correlation profiling in OP

**FIGURE 6:**
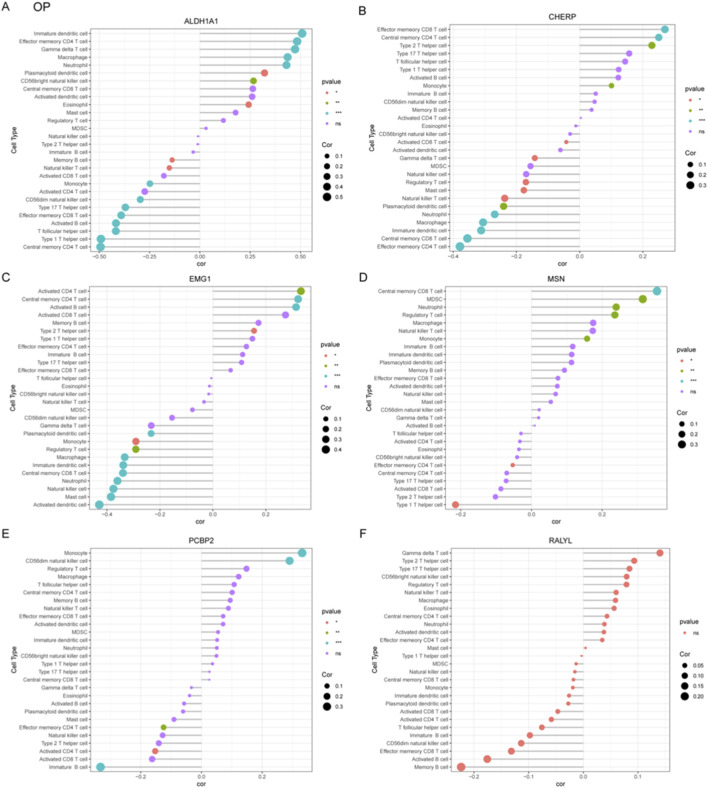
Correlation analysis between OP-associated gene signatures and immune cell subtypes. **(A–F)** Correlation coefficients were calculated to explore the relationships between six candidate diagnostic genes including ALDH1A1 **(A)**, CHERP **(B)**, EMG1 **(C)**, MSN **(D)**, PCBP2 **(E)**, and RALYL **(F)** and infiltrating immune cell populations in OP.

To systematically characterize immune-related gene signatures in OP, correlation analyses were performed between six candidate genes (ALDH1A1, CHERP, EMG1, MSN, PCBP2, RALYL) and various immune cell populations. ALDH1A1 exhibited significant negative correlation with immature dendritic cell and positive correlation with central memory CD4^+^ T cell (Figure 6A). CHERP showed negative correlation with effector memory CD8^+^ T cell and positive correlation with effector memory CD4^+^ T cell (Figure 6B). EMG1 demonstrated negative correlation with activated CD4^+^ T cell but positive association with activated dendritic cell (Figure 6C). The MSN gene displayed negative correlation with central memory CD8^+^ T cells but positive association with type I T helper cell (Figure 6D). PCBP2 showed positive relationship with immature B cell but negative correlation with monocyte (Figure 6E). RALYL did not show significant correlation with these immune cells (Figure 6F). These results implicate dysregulated T cell differentiation and innate immune activation as potential drivers of OP progression, warranting mechanistic validation in targeted studies.

### Correlation profiling of immune-related genes and cellular signatures in CKD and OP

**FIGURE 7:**
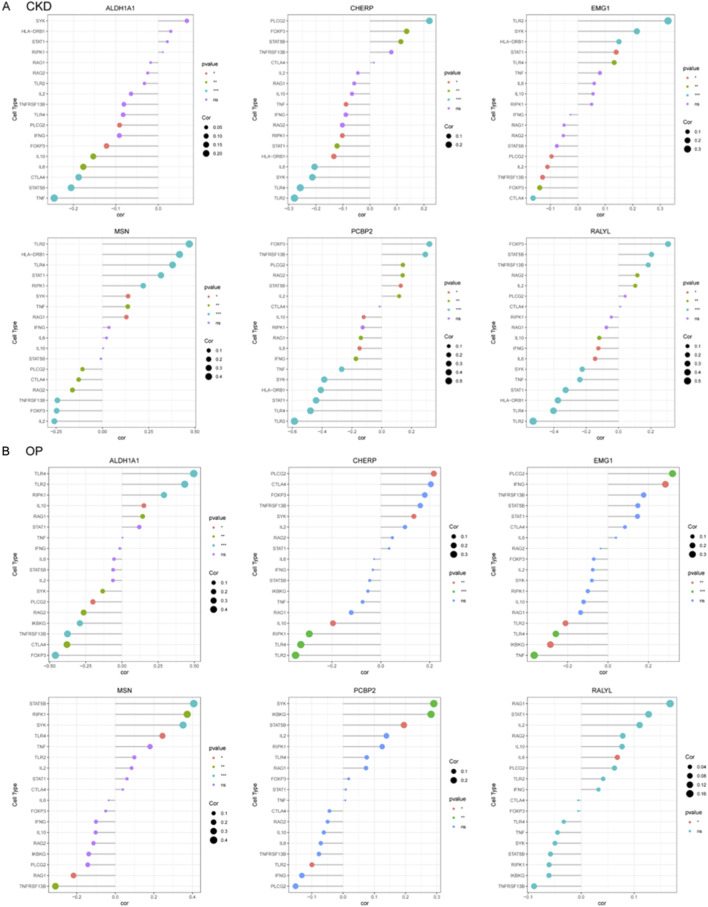
Correlation profiling of diagnostic genes with immune and inflammatory cell markers in CKD and OP. **(A)** Correlation analysis between six core genes (ALDH1A1, CHERP, EMG1, MSN, PCBP2, and RALYL) and immune-related markers in CKD. **(B)** In OP, correlation analysis between six core genes (ALDH1A1, CHERP, EMG1, MSN, PCBP2, and RALYL) and immune-related markers was presented.

To systematically investigate immune-gene interactions in CKD, correlation analyses were performed between the key genes (ALDH1A1, CHERP, EMG1, MSN, PCBP2, RALYL) and immune/inflammatory cell markers (Figure 7A). ALDH1A1 exhibited significant positive correlations with pro-inflammatory markers TNF. CHERP, PCBP2, and RALYL showed strong positive correlation withs TLR2 and TLR4. EMG1 and MSN were significantly negative with TLR2.

The correlation analyses in OP were also performed between the key candidate genes and immune/inflammatory cell markers (Figure 7B). ALDH1A1, MSN, and RALYL exhibited significant positive correlations with TNFRSF13B. CHERP and EMG1 showed significantly negative correlation with PLCG2, which was positively correlated with PCBP2.

### CKD exacerbates estrogen deficiency-induced bone loss and osteoclast activation

**FIGURE 8:**
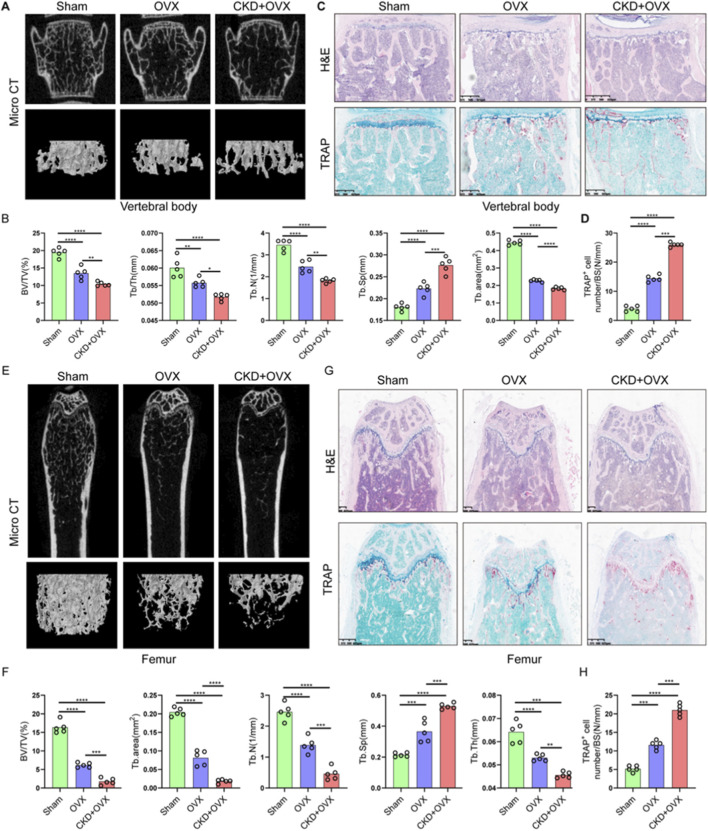
CKD synergistically exacerbates ovariectomy (OVX)-induced trabecular bone loss and osteoclast activation in mice. **(A)** Representative micro-CT images of lumbar vertebral trabecular bone from Sham, OVX, and CKD + OVX mice. **(B)** Quantitative micro-CT analyses of vertebral bone parameters. **(C)** H&E staining of vertebral sections corroborates structural degeneration. **(D)** TRAP staining indicates increased osteoclast number per bone surface in CKD + OVX group, reflecting enhanced osteoclastic activity. **(E)** Representative micro-CT images of femoral trabeculae. **(F)** Quantitative micro-CT analyses of femoral bone. **(G)** H&E-stained femoral sections show trabecular bone loss consistent with imaging results. **(H)** TRAP staining of femur. Data represent mean ± SD (n = 5 mice per group). *p < 0.05, **p < 0.01, ***p < 0.001, ****p < 0.0001, ns = not significant (one-way ANOVA).

To investigate the impact of CKD on osteoporosis progression, we established a combined CKD and ovariectomy (CKD + OVX) mouse model and performed comprehensive microstructural and histological assessments of vertebral and femoral bones. Micro-CT analysis of lumbar vertebrae revealed a marked deterioration of trabecular architecture in CKD + OVX mice, compared with Sham and OVX groups (Figure 8A). Quantitative analysis showed a significant reduction in bone volume fraction (BV/TV), trabecular thickness (Tb/Th), trabecular number (Tb.N), and Tb. area, accompanied by an increase in trabecular separation (Tb.Sp) in OVX mice, with these parameters further exacerbated in CKD + OVX animals (Figure 8B). Histological examination with H&E staining corroborated these findings, demonstrating notable trabecular bone loss in the CKD + OVX group (Figure 8C). Furthermore, TRAP staining revealed increased osteoclast number in the CKD + OVX group, as indicated by a significantly elevated number of TRAP^+^ cells per bone surface (Figure 8D), suggesting heightened osteoclastic activity. A similar pattern of deterioration was observed in the femoral bone. Micro-CT analysis demonstrated that CKD + OVX mice exhibited the most severe disruption of trabecular structure (Figure 8E), reflected by further reductions in BV/TV, Tb. area, Tb.Th, and Tb.N, along with a significant increase in Tb. Sp (Figure 8F). These microstructural deficits were also supported by H&E staining of femoral trabeculae (Figure 8G). Notably, TRAP staining revealed a pronounced elevation in osteoclast numbers in CKD + OVX mice compared with Sham and OVX groups (Figure 8H), further confirming the synergistic promotion of bone resorption under CKD condition.

Collectively, these findings demonstrate that CKD markedly worsens the osteoporotic phenotype induced by estrogen deficiency, promoting a severe imbalance in bone remodeling characterized by trabecular bone loss and enhanced osteoclastogenesis.

### CKD-induced renal fibrosis and OP-Associated bone loss share dysregulation of osteocyte-related gene

**FIGURE 9:**
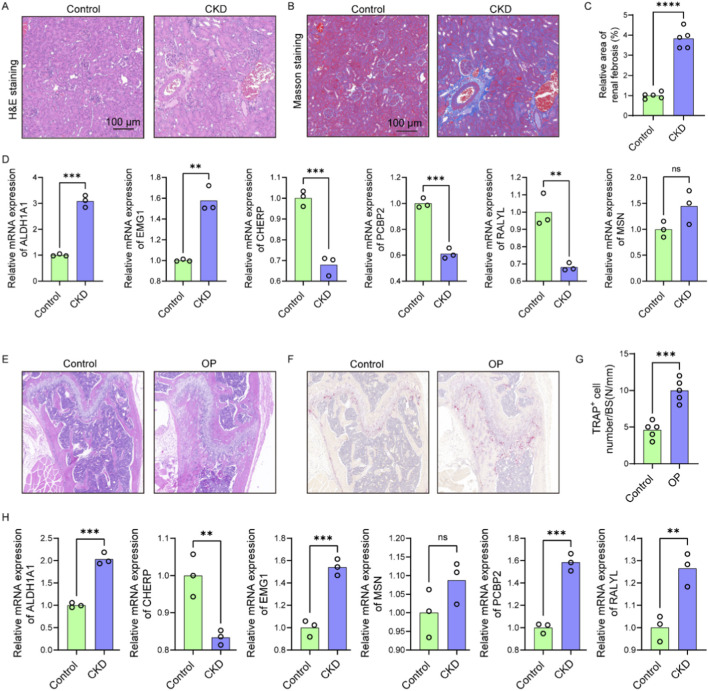
CKD-induced renal fibrosis and OP-associated bone loss exhibit shared dysregulation of osteocyte-related genes. **(A)** Representative H&E-stained images of kidney sections from control and CKD mice showing tubular dilation, epithelial atrophy, and inflammatory infiltration (n = 5). **(B)** Masson’s trichrome staining of renal sections indicating increased collagen deposition in CKD mice (n = 5). **(C)** Quantification of fibrotic area based on Masson staining (n = 5). **(D)** Relative mRNA expression levels of six candidate genes (ALDH1A1, EMG1, CHERP, PCBP2, RALYL, and MSN) in kidney tissues of control and CKD mice (n = 3). **(E)** H&E staining of femoral bone sections showing trabecular structure disruption and reduced bone mass in OP mice compared to controls (n = 5). **(F)** Representative TRAP staining images of femoral sections from control and OP mice (n = 5). **(G)** Quantification of TRAP^+^ osteoclast number per bone surface (n = 5). **(H)** Expression levels of the six candidate genes in femoral bone tissues of control and OP mice (n = 3). Scale bars: 100 μm. Data represent mean ± SD. *p < 0.05, **p < 0.01, ***p < 0.001, ****p < 0.0001, ns = not significant.

To validate the functional relevance of candidate genes identified through bioinformatic analysis, 5/6 nephrectomy mice was used to mimic CKD condition. H&E staining of kidney tissues revealed significant tubular dilation, epithelial atrophy, and inflammatory cell infiltration in CKD mice compared to controls (Figure 9A). Masson staining further confirmed increased collagen deposition within the renal interstitium (Figure 9B), and quantitative analysis demonstrated a significant increase in fibrotic area (Figure 9C), consistent with tubulointerstitial fibrosis. Gene expression analysis of renal tissues showed significant upregulation of ALDH1A1 and EMG1, while CHERP, PCBP2, and RALYL were markedly downregulated in CKD kidneys compared with controls (Figure 9D). Although MSN level was slightly raised in the CKD kidneys, no significant difference was observed between the two groups (Figure 9D). These results confirm that CKD alters the expression of multiple genes implicated in lactylation and osteocyte function, potentially linking renal dysfunction to systemic bone pathology.

We further examined trabecular bone morphology in an OP mouse model. H&E staining of femoral bone sections from OP mice revealed disrupted trabecular architecture and reduced bone mass compared to control mice (Figure 9E). TRAP staining showed increased numbers of TRAP^+^ multinucleated osteoclasts (Figure 9F), with quantification confirming a significant elevation in osteoclast number per bone surface in OP mice (Figure 9G), indicative of enhanced bone resorption. Expression analysis in OP bone tissues showed significantly elevated levels of ALDH1A1, EMG1, PCBP2, and RALYL, while CHERP was significantly reduced and MSN level was slightly raised (Figure 9H). According to the above findings, the expression models of ALDH1A1, EMG1, and CHERP were consistent between the OP bone tissue and the CKD renal tissue, underscoring the shared molecular features between CKD-induced and primary osteoporosis. These data reinforce the role of CKD as a driver of skeletal deterioration and support the diagnostic relevance of the identified hub genes in mediating renal–skeletal crosstalk.

## Discussion

The primary aim of this study was to elucidate the potential molecular mechanisms linking CKD and OP by leveraging bioinformatics tools and lactylation-related gene profiling. Our key findings offer significant insights into the pathophysiological interactions between these two diseases, identifying novel biomarkers and constructing predictive models that may inform both diagnosis and treatment strategies.

The identification of 15 common DEGs shared between CKD, OP, and lactylation pathways is a crucial outcome of this study, which represents a core set of candidates for mediating the CKD-OP comorbidity through metabolic reprogramming. While the functional roles of this specific gene set in the context of lactylation are novel, several genes have established connections to relevant cellular processes. For instance, ALDH1A1 was involved in oxidative stress response and retinoic acid synthesis, both critical in bone remodeling and renal fibrosis (Han et al., 2025). MSN (Moesin) and EMG1 (ER Membrane Protein Complex Subunit 1) were implicated in cell adhesion and mitochondrial function, which processed fundamental to both osteocyte and podocyte function (Rahimi et al., 2021; Couto-Lima et al., 2024). The presence of RNA-binding proteins like PCBP2 and RALYL suggested a potential layer of post-transcriptional regulation that may be influenced by the metabolic state (Qi et al., 2025; Schreiberhub et al., 2024). Although the functional connections for all 15 genes in this specific triad have not yet been fully characterized, these genes above provide a foundation for understanding the molecular convergence of CKD and OP, particularly through the lens of lactylation, a post-translational modification that has not been extensively studied in these contexts (Li et al., 2022). Recent research has highlighted the increasing importance of lactylation in various pathological conditions, particularly in metabolic diseases, where it has been linked to altered gene expression and metabolic dysregulation (Wang et al., 2022a). However, its role in CKD and OP has largely remained unexplored. Our results suggest that lactylation could play a pivotal role in the shared pathophysiology of these diseases, aligning with emerging studies that have begun to investigate the involvement of lactylation in skeletal and renal disorders. This discovery opens the door to potential therapeutic targets for intervention, offering a novel approach to managing the interplay between CKD and OP. For instance, recent work on lactate metabolism has shown that lactate, and its associated modifications like lactylation, can influence the inflammatory pathways that are central to both CKD and OP (Wang et al., 2024a; Huang et al., 2024). This emphasizes the need for further research into lactylation as a modulator of disease progression in both kidney dysfunction and bone loss.

Furthermore, the construction of robust diagnostic models using machine learning techniques, which exhibited high AUC values across independent cohorts, underscores the potential of these models for clinical application. The high AUC values not only demonstrate the accuracy of the models in distinguishing between CKD and OP but also suggest that these predictive tools could be employed as reliable diagnostic aids in clinical settings. Recent advances in bioinformatics and machine learning have significantly improved the predictive accuracy of diagnostic models, with studies integrating large-scale omics data to better understand complex diseases like CKD and OP (Lee et al., 2022; Lo et al., 2024). The ability to integrate bioinformatics data with machine learning to produce such diagnostic models represents a significant advancement in precision medicine, building on current efforts that have utilized machine learning for biomarker discovery and disease prediction in chronic conditions. Our results contribute to this trend by demonstrating the clinical relevance of machine learning models that incorporate multi-dimensional data, offering a promising tool for early diagnosis and monitoring of CKD and OP.

The discovery and validation of six hub genes, MSN, PCBP2, CHERP, EMG1, RALYL, and ALDH1A1, implicated in both CKD and OP is another noteworthy contribution of this study. The other nine genes from the shared list, while interesting, did not meet this stringent set of criteria across all analyses. These genes, identified through stringent bioinformatics analysis, serve as potential biomarkers for both diseases. Their dual involvement in CKD and OP may reflect shared molecular pathways, such as inflammation, bone mineralization, and renal dysfunction, that exacerbate disease progression. The involvement of inflammation in CKD and OP has been well-documented in recent literature, with several studies highlighting how chronic inflammation in kidney disease accelerates bone loss through dysregulated cytokine release and impaired osteoblast function (Ren et al., 2018; Yang et al., 2025). Similarly, the role of oxidative stress and mitochondrial dysfunction in both conditions is increasingly recognized as a common mechanism driving disease progression (Rodríguez-Sánchez et al., 2021). Recent research has also shown that ALDH1A1, one of the hub genes identified in this study, is crucial in regulating oxidative stress and maintaining cellular homeostasis (Calleja et al., 2021; Bellala et al., 2024), reinforcing its potential as a therapeutic target. It is noteworthy that the expression trend of several genes, such as PCBP2, differed between CKD kidney and OP bone tissues, being downregulated in the former and upregulated in the latter. While seemingly contradictory, this discrepancy likely reflects the distinct tissue-specific and cell-type-specific pathological contexts. In CKD, the dominant processes of fibrosis and inflammation may engage regulatory networks that suppress PCBP2 expression, potentially as part of a maladaptive response. Conversely, in the OP bone microenvironment, the heightened bone resorption and altered cellular stress responses might drive an increase in PCBP2, which could play a different role in post-transcriptional regulation relevant to osteoclast or osteoblast activity. This divergence underscores that the functional consequence of a shared gene signature can be context-dependent, influenced by the unique constellation of cell types and signaling pathways active in each diseased tissue. Future single-cell studies and cell-type-specific functional experiments will be crucial to dissect these complex, tissue-specific roles. Targeting these hub genes could open avenues for therapeutic interventions aimed at mitigating the impact of CKD on bone health, potentially slowing the progression of OP in CKD patients. The growing body of research on these genes underscores their relevance in the pathophysiology of CKD and OP, and their validation in our study provides a strong basis for future investigation into their role as therapeutic targets.

In addition to identifying key biomarkers, we developed accurate nomogram models for risk prediction in both CKD and OP. These nomograms, which incorporate multiple variables, offer a practical tool for clinicians to assess individual risk profiles in patients suffering from either condition. By combining clinical data with the molecular insights gleaned from our study, these nomograms can provide a personalized approach to patient care, facilitating earlier detection and more tailored treatment strategies. The growing use of nomograms in medical practice has been a significant advancement in precision medicine, particularly in the management of complex, multifactorial diseases like CKD and OP (Wang et al., 2024b; Zhuo et al., 2024). Recent studies have demonstrated the utility of nomograms in predicting disease progression and treatment outcomes, particularly in oncology and cardiovascular diseases (Wu et al., 2020; Wang et al., 2022b). Notably, the differential generalizability observed between the CKD and OP nomograms, with the former demonstrating robust performance and the latter showing limited external validation, likely stems from fundamental differences in disease pathophysiology and dataset characteristics. The CKD model benefited from transcriptomic data derived from a defined tissue source, whereas the OP signature was identified from peripheral blood monocytes and B cells, which could be subject to considerable systemic biological noise beyond bone-specific metabolism. Furthermore, the OP model aimed to distinguish individuals based on a continuous trait (low vs. high BMD) within an etiologically heterogeneous group, complicating the identification of a universal transcriptional signature that transfers robustly across independent cohorts. Thus, while the limited generalizability of the OP model underscores the challenge in defining a non-invasive, blood-based biomarker for a multifactorial disorder like osteoporosis, it does not invalidate the shared lactylation-related biology central to this study. Furthermore, the integration of multi-omics data into nomogram models, as shown in our study, is a cutting-edge approach that enhances their predictive power. Current research is also focused on refining these models by incorporating novel biomarkers and clinical variables, a trend that aligns with our work and reinforces the potential of nomograms to transform clinical decision-making. This evolving application of nomograms reflects the increasing importance of personalized medicine, offering clinicians a dynamic tool for improving patient care and outcomes.

Our immune correlation analyses revealed several compelling associations that align with established disease biology. For instance, in CKD, the strong positive correlation between PCBP2, Th 17 T helper cell (Figure 5E) is particularly intriguing. Given the documented role of PCBP2 in regulating T cell function gene, Foxp3, this correlation suggests a potential mechanism by which PCBP2 could contribute to the sustained adaptive immune activation characteristic of CKD progression (Figure 7A). In OP, the positive correlation of MSN with macrophages (Figure 6D) resonates with the established paradigm of pro-inflammatory macrophages skewing in bone loss, as the MSN protein is involved in cytoskeletal organization and immune cell migration, potentially facilitating Th1 recruitment to bone sites. These specific, strong correlations move beyond mere association and provide a grounded, hypothesis-generating framework for future mechanistic studies aimed at understanding how these lactylation-related genes directly interface with the immune system to drive pathology in CKD and OP.

Importantly, our findings highlight that CKD exacerbates the progression of OP, which is consistent with previous reports that chronic kidney dysfunction can compromise bone metabolism (Biruete et al., 2020; Allen et al., 2015), leading to increased bone fragility and higher fracture risk. The synergistic bone loss observed in our CKD + OVX mouse model can be plausibly linked to the functional roles of the identified hub genes. For instance, the significant upregulation of ALDH1A1 in both CKD kidney and OP bone tissue may represent a compensatory response to severe oxidative stress, a common feature of both CKD and estrogen deficiency. However, a maladaptive, chronic upregulation could paradoxically fuel pathogenic processes, such as promoting the differentiation and survival of hyper-resorptive osteoclasts, thereby exacerbating the trabecular bone loss seen in the CKD + OVX group. Concurrently, the downregulation of CHERP, a key regulator of intracellular calcium homeostasis, may provide another compelling link (Yamanaka et al., 2022). In the context of CKD-MBD, systemic calcium-phosphate imbalances are compounded at the cellular level. Dysregulated CHERP expression in osteoblasts or osteoclasts could disrupt precise calcium signaling, which is critical for osteoclast resorption activity and osteoblast mineralization. This dual dysregulation, where ALDH1A1 upregulation may drive excessive osteoclast activity and CHERP downregulation impairs bone formation, offers a plausible molecular explanation for the severely disrupted bone remodeling equilibrium and the exacerbated osteoporotic phenotype in comorbid conditions. Recent research has further expanded our understanding of these mechanisms, particularly the role of mineral and bone disorders in CKD, which have been shown to influence bone mineralization and fracture risk (Pazianas and Miller, 2021). Studies have indicated that dysregulation of phosphate, calcium, and vitamin D metabolism, often seen in CKD, contributes to the deterioration of bone quality and density, thus increasing the risk of OP (Hu et al., 2022). Moreover, the inflammatory response in CKD, characterized by elevated levels of cytokines and pro-inflammatory mediators, has been implicated in both bone resorption and impaired bone formation, further accelerating bone loss (Mazzaferro et al., 2020). The emerging recognition of the gut-kidney-bone axis in CKD-related bone diseases is another important area of current research, with studies showing that gut microbiota dysbiosis may exacerbate bone loss (Rodrigues et al., 2021; Kermgard et al., 2021). Our study contributes to this growing body of evidence by providing a molecular framework through which CKD aggravates OP, particularly by implicating lactylation and other key molecular pathways. This molecular understanding not only sheds light on the pathophysiology of CKD-related OP but also offers new opportunities for therapeutic interventions targeting specific molecular pathways that could mitigate the progression of both diseases.

This study has several limitations. First, the connection between lactylation and the identified hub genes remains speculative, as our bioinformatic approach lacks direct experimental validation. To definitively establish lactylation as a functional regulator, future studies must employ site-specific lactylome profiling and functional assays in disease models to confirm its mechanistic role in governing these shared pathways. Second, biases may exist in the bioinformatics analysis due to the reliance on publicly available data, which could impact the accuracy and completeness of the results. Third, while the performance of our diagnostic models was excellent on internal and independent validation cohorts, such as CKD, a direct comparison with existing diagnostic models was not feasible in this study. The lack of a standardized benchmark dataset incorporating both clinical and transcriptomic data prevents a definitive head-to-head evaluation of whether our gene signature provides additive value over established clinical parameters alone. Future prospective studies designed to collect multi-modal data are essential to validate the comparative and incremental utility of our model in a real-world clinical setting. Forth, while our study identified a compelling association between shared lactylation-related gene signatures and the comorbidity of CKD and OP, the precise mechanistic role of lactylation in regulating these hub genes remains speculative. Our current bioinformatic approach, while powerful for identification, does not elucidate whether the identified genes are drivers or consequences of the altered lactylation landscape, nor does it determine if they are direct targets of lactylation or are indirectly regulated through lactylation-dependent transcriptional programs. The causal relationships and the exact functional impact of lactylation on the activity of genes such as CHERP, PCBP2, and EMC1 in the context of renal and bone pathology are still unknown. Therefore, future functional studies are essential to directly validate these hypotheses. This should include site-specific mapping of lactylation marks on these proteins, coupled with gain- and loss-of-function experiments in relevant cell types, such as osteoblasts, osteoclasts, and renal tubular cells, to definitively establish lactylation as a functional bridge regulating these shared pathways in CKD and OP. Finally, the mechanistic link whereby CKD exacerbates bone loss through lactylation remains undefined, necessitating functional validation via targeted interventions. Addressing these limitations in future research will be essential to confirm and expand upon the findings of this study.

In conclusion, our study offers important insights into the molecular mechanisms that link CKD and OP, providing a deeper understanding of how lactylation and specific gene networks contribute to the co-morbidity of these diseases. The development of diagnostic models and nomograms paves the way for improved clinical management of CKD and OP patients, emphasizing the need for integrated approaches that address both renal and skeletal health. Future research should aim to further validate the identified biomarkers and explore the therapeutic potential of targeting lactylation-related pathways in CKD and OP.

## Data Availability

The original contributions presented in the study are included in the article/Supplementary Material, further inquiries can be directed to the corresponding authors.
