# Supplementary material for: Identification of shared lactylation-related gene signatures between osteoporosis and chronic kidney disease
Source: Front Cell Dev Biol. 2025 Dec 11;13:1719273. doi: 10.3389/fcell.2025.1719273 (PMC12738852; doi:10.3389/fcell.2025.1719273)
Supplement: Supplementary file 1 [file DataSheet1.docx]

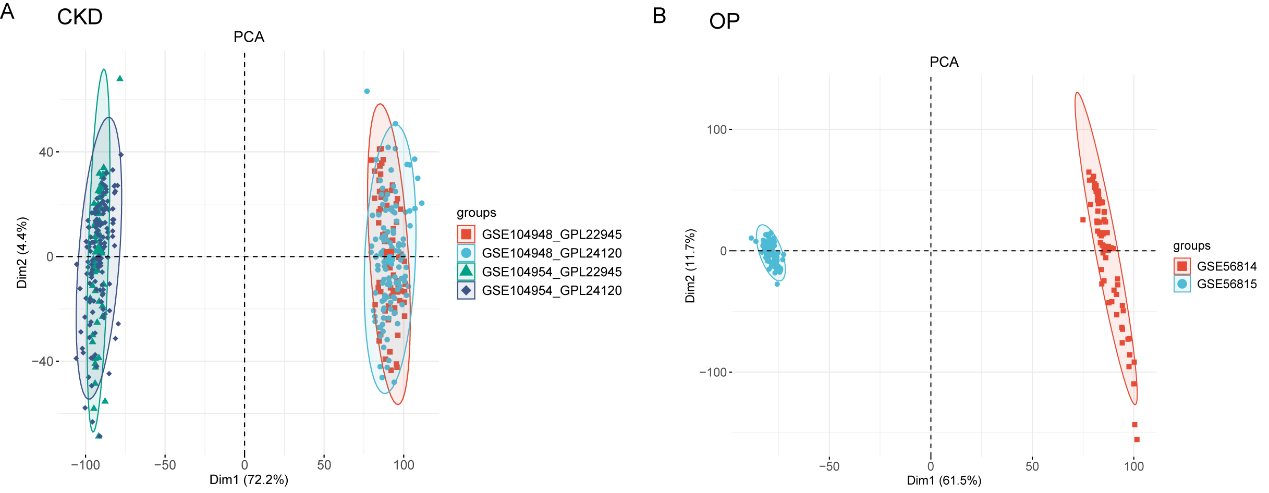


**Figure S1** PCA analysis for CKD (A) and OP (B), respectively.


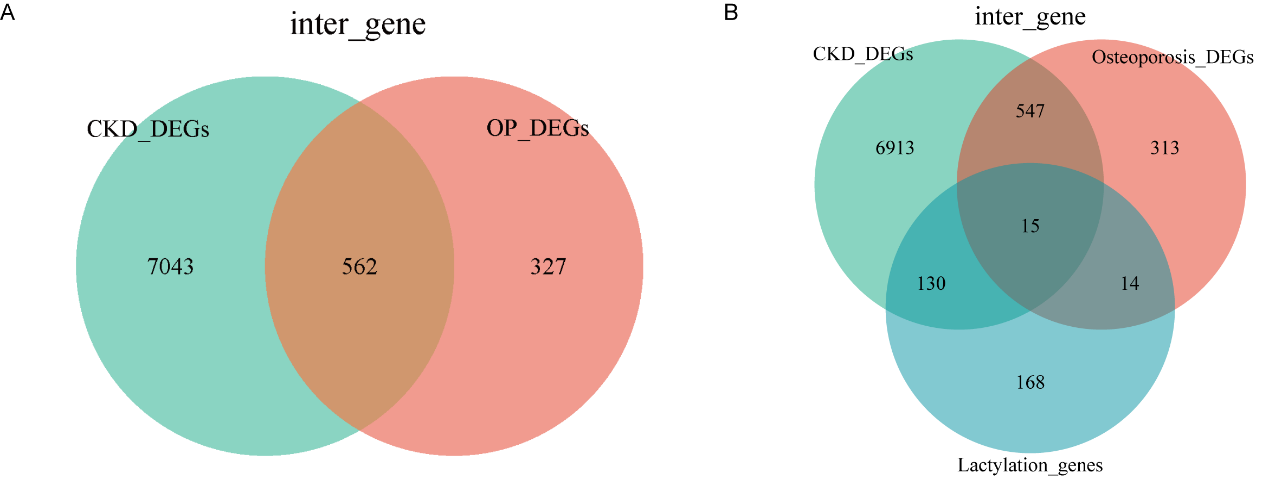


**Figure S2** Venn diagrams delineated molecular intersections between CKD and OP pathogenesis. A total of 562 differentially expressed genes (DEGs) were shared between CKD and OP, while 327 and 7043 genes were unique to OP and CKD, respectively (Fig. Sup 2A).

| PCBP2 |
| --- |
| CCT5 |
| EIF3D |
| MSN |
| CD2BP2 |
| SET |
| EMG1 |
| CHERP |
| PSMA7 |
| RALYL |
| S100A11 |
| ALDH1A1 |
| PRDX1 |
| TERF2 |
| RPA1 |

**Supplementary Table 1** List of the 15 shared genes across CKD_DEGs, OP_DEGs, and lactylation-related genes
